# Analysis of Heat Transfers inside Counterflow Plate Heat Exchanger Augmented by an Auxiliary Fluid Flow

**DOI:** 10.1155/2014/308545

**Published:** 2014-02-25

**Authors:** A.-R. A. Khaled

**Affiliations:** Mechanical Engineering Department, King Abdulaziz University, P.O. Box 80204, Jeddah 21589, Saudi Arabia

## Abstract

Enhancement of heat transfers in counterflow plate heat exchanger due to presence of an intermediate auxiliary fluid flow is investigated. The intermediate auxiliary channel is supported by transverse conducting pins. The momentum and energy equations for the primary fluids are solved numerically and validated against a derived approximate analytical solution. A parametric study including the effect of the various plate heat exchanger, and auxiliary channel dimensionless parameters is conducted. Different enhancement performance indicators are computed. The various trends of parameters that can better enhance heat transfer rates above those for the conventional plate heat exchanger are identified. Large enhancement factors are obtained under fully developed flow conditions. The maximum enhancement factors can be increased by above 8.0- and 5.0-fold for the step and exponential distributions of the pins, respectively. Finally, counterflow plate heat exchangers with auxiliary fluid flows are recommended over the typical ones if these flows can be provided with the least cost.

## 1. Introduction

Counterflow plate heat exchangers are widely used in various engineering applications especially preheat, chemical, pharmaceutical, and food processing applications [1]. This is because both hot and cold fluids within the plate heat exchanger are exposed to a much larger surface area per unit volume than that in the conventional (double pipe) heat exchanger [2]. Also, plate heat exchangers can have hydraulic diameters smaller than 2 mm. This can lead to having larger heat transfer coefficients. Thus, plate heat exchangers have larger effectiveness compared to conventional counterflow heat exchangers. Additionally, many of the passive heat transfer enhancement tools like fins and rough surfaces [3–5] can easily be installed in the plate heat exchanger as compared to the conventional heat exchanger. This is why finned plate heat exchangers [6] and gasketed plate heat exchangers [7] are widely spread in many industrial applications.

The most recent literature reviews on passive heat transfer enhancements in heat exchangers [8, 9] show that the major analyzed enhancement methods are the following: (1) twisted tape, (2) wire coil, (3) swirl flow, (4) conical ring, and (5) ribs. All of these devices augment heat transfer because they tend to disturb the fluid flows [3, 10]. Therefore, it can be concluded that enhancing heat transfer in plate heat exchangers under laminar flow conditions did not receive much attention by researchers. Perhaps the most recent proposal for heat transfer enhancement in heat exchangers under laminar flow conditions is the use of nanofluids [11–13]. However, not all nanofluids can be adequate for processing special products like pharmaceutical and food products. This is because the commonly used nanoparticles can be harmful to human body [14, 15]. Consequently, the present work aims to propose and analyze a new method for enhancing heat transfer in plate heat exchanger without altering either the velocity profiles or compositions of both hot and cold fluids.

The proposed plate heat exchanger is composed of hot and cold fluid channels separated by an auxiliary fluid channel. This auxiliary channel may contain as many passive enhancement tools as possible. Accordingly, both the velocity profile and the composition of the hot and cold fluids are preserved. The heat transfer enhancement in the proposed system is due to the following combined effects: (1) convection of the auxiliary fluid and (2) passive enhancement mechanisms in the auxiliary channel. In the present work, transverse pins connecting the facing boundaries of both hot and cold fluid channels are considered as one of passive enhancement mechanisms [16, 17]. Moreover, the auxiliary fluid is considered to flow in the direction cross to both hot and cold fluid flow directions. Accordingly, the auxiliary channel length (hot/cold channels width) can be selected to be small enough to have boundary layer flows [18, 19]. Hence, convection thermal resistances between the auxiliary fluid and both hot and cold fluids are minimized. The heat transfer rates within the present system are expected to be higher than those in conventional system for specific auxiliary flow conditions. Accordingly, the present work additionally aims to identify some trends of parameters that cause enhancement ratios to be above unity. Modeling laminar flow and heat transfer inside two dimensional channels including auxiliary channels is well established in the literature [18–22].

In the present work, heat transfer inside plate heat exchanger with auxiliary fluid channel separating the hot and cold fluid channels is modeled and analyzed. Both hot and cold fluid flows are considered to be laminar under hydrodynamically fully developed condition. The energy equations of the hot and cold fluids are coupled with the energy equations of the auxiliary fluid boundary layers. The solution of the momentum and energy equations within the boundary layers is well established [18–20]. Accordingly, both coupled hot and cold fluid energy equations are solved numerically using finite difference methods. Approximate analytical solutions for the heat transfer rates under the fully developed flow and very long pins conditions are derived. A number of heat transfer performance ratios including the heat exchanger effectiveness ratios are computed. A parametric study for heat transfer enhancement is made to recognize the conditions of controlling parameters that produce favorable enhancement factors.

## 2. Problem Formulation

### 2.1. Modeling of Flow and Heat Transfer inside the Hot and Cold Fluid Channels

**Figure 1:**
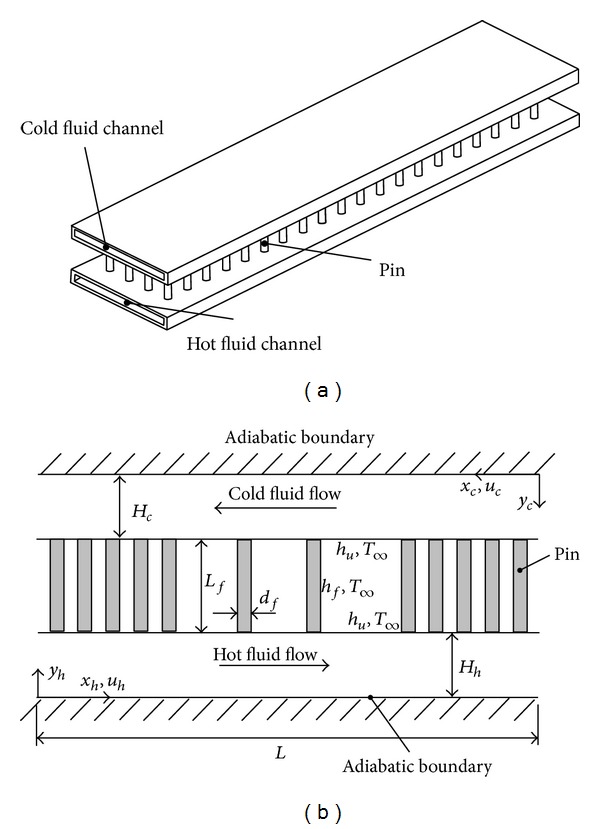
(a) 3D view of the counterflow plate heat exchanger with intermediate auxiliary channel and (b) schematic profile of the device and the coordinates system.

Consider two parallel channels of length *L* and width *W*. The first channel confines the hot fluid flow while the second one contains the cold fluid flow. The solid boundaries of these channels facing each other are perfectly connected together via cylindrical pins of diameter *d*_*f*_ and length *L*_*f*_ as shown in Figure 1. These pins are surrounded by an auxiliary fluid stream of free stream temperature *T*_*∞*_ and convection heat transfer coefficient *h*_*f*_. The convection heat transfer coefficient between the auxiliary fluid stream and the channel boundaries facing that stream is *h*_*u*_. Accordingly, heat transfers between the channels and the auxiliary fluid are due to convection over the unfinned surfaces and conduction via the connecting pins. In contrast, the outermost solid boundaries of the heat exchanger are considered to be adiabatic so that heat transfer rates in the heat exchanger are maximized.

The dimensionless momentum and energy equations of the hot and cold fluids are [21]

(1) $$\begin{array}{c} \frac{d^{2}U_{h,c}}{dY_{h,c}^{2}}=-12, \end{array}$$

(2) $$\begin{array}{c} a_{h,c}{\text{Pe}}_{h,c}U_{h,c}\frac{\partial \theta_{h,c}}{\partial X_{h,c}}=\frac{\partial^{2}\theta_{h,c}}{\partial Y_{h,c}^{2}}, \end{array}$$

where *U*_*h*_ and *U*_*c*_ are the dimensionless axial velocity fields for the hot and cold fluids, respectively. *θ*_*h*_ and *θ*_*c*_ are the hot and cold fluid dimensionless temperatures, respectively. *X*_*h*_ and *X*_*c*_ are the dimensionless axial positions of the hot and cold fluids, respectively. *Y*_*h*_ and *Y*_*c*_ are the dimensionless transverse positions of the hot and cold fluids, respectively. The channels aspect ratios *a*_*h*,*c*_ as well as the hot and cold flow Péclet numbers Pe_*h*_ and Pe_*c*_ are given by

(3) $$\begin{array}{c} a_{h,c}=\frac{H_{h,c}}{L}, \end{array}$$

(4) $$\begin{array}{c} {\text{Pe}}_{h,c}=\frac{\rho_{h,c}c_{ph,pc}u_{mh,mc}H_{h,c}}{k_{h,c}}, \end{array}$$

where *H*_*h*_ and *H*_*c*_ are the heights of the hot and cold fluid channels, respectively. (*ρ*_*h*_, *ρ*_*c*_), (*c*_*ph*_, *c*_*pc*_), and (*k*_*h*_, *k*_*c*_) are the density, specific heat, and thermal conductivity pairs of the hot and cold fluids, respectively. *u*_*mh*_ and *u*_*mc*_ are the mean axial velocities of the hot and cold fluids, respectively.

The dimensionless variables given in (1) and (2) are defined as

(5a) Xh,c=xh,cL;  Yh,c=yh,cHh,c;

(5b) $$\begin{array}{c} U_{h,c}=\frac{u_{h,c}}{u_{mh,mc}}; \end{array}$$

(5c) $$\begin{array}{c} \theta_{h}\left(X_{h},Y_{h}\right)=\frac{T_{h}\left(x_{h},y_{h}\right)-T_{h1}}{T_{c1}-T_{h1}}; \end{array}$$

where *x*_*h*_ and *y*_*h*_ are the axial and transverse positions of the hot fluid, respectively. *x*_*c*_ and *y*_*c*_ are the corresponding positions of the cold fluid, respectively. *x*_*h*_ and *x*_*c*_ start from zero at the fluid inlets while *y*_*h*_ and *y*_*c*_ start from zero at the adiabatic boundaries. *u*_*h*_ and *u*_*c*_ are the axial velocity fields for the hot and cold fluids, respectively. *T*_*h*_ and *T*_*c*_ are the hot and cold fluid temperatures, respectively. *T*_*h*1_ and *T*_*c*1_ are the inlet temperatures of the hot and cold fluids, respectively.

Equation (1) is based on hydrodynamically fully developed conditions. Their solutions under no-slip conditions at the boundaries are given as [21]

(6) $$\begin{array}{c} U_{h,c}=6Y_{h,c}\left(1-Y_{h,c}\right). \end{array}$$

The boundary conditions of (2) are given by

(7a) $$\begin{array}{c} \theta_{h,c}\left(X_{h,c}=0,Y_{h,c}\right)=0, \end{array}$$

(7b) $$\begin{array}{c} {\frac{\partial \theta_{h,c}}{\partial Y_{h,c}}|}_{Y_{h,c}=0}=0, \end{array}$$

(7c) $$\begin{array}{c} {\frac{\partial \theta_{h}}{\partial Y_{h}}|}_{Y_{h}=1}=\frac{\phi q_{fh}^{\prime \prime }+\left(1-\phi \right)q_{uh}^{\prime \prime }}{k_{h}\left(T_{h1}-T_{c1}\right)/H_{h}}, \end{array}$$

(7d) $$\begin{array}{c} -{\frac{\partial \theta_{c}}{\partial Y_{c}}|}_{Y_{c}=1}=\frac{\phi q_{fc}^{\prime \prime }+\left(1-\phi \right)q_{uc}^{\prime \prime }}{k_{c}\left(T_{h1}-T_{c1}\right)/H_{c}}, \end{array}$$

where *q*_*fh*_′′ and *q*_*fc*_′′ are the conduction heat fluxes through the pin bases at the hot and cold surfaces, respectively. However, *q*_*uh*_′′ and *q*_*uc*_′′ are the convection heat fluxes at the unfinned portions of the hot and cold surfaces, respectively. The local pins base area concentration denoted by *ϕ* can be calculated from the following expression:

(8) $$\begin{array}{c} \phi =\frac{\pi d_{f}^{2}}{4W}\left(\frac{dN_{f}}{dx}\right), \end{array}$$

where *dN*_*f*_/*dx* is the local axial gradient of the number of pins (*N*_*f*_).

### 2.2. Modeling of the Pins Conduction and Auxiliary Fluid Convection Heat Fluxes

The one-dimensional fin equation [18, 19] can be used to model the conduction heat transfer through the pins. This fin equation has the following dimensionless form:

(9) $$\begin{array}{c} \frac{d^{2}\theta_{f}}{dX_{f}^{2}}-{\left(m^{*}\right)}^{2}\theta_{f}=0, \end{array}$$

where the dimensionless pin distance, *X*_*f*_, the dimensionless pin local temperature, *θ*_*f*_, and the dimensionless pin thermal length, m*, are given by

(10a) $$\begin{array}{c} X_{f}=\frac{x_{f}}{L_{f}}; \end{array}$$

(10b) $$\begin{array}{c} \theta_{f}\left(X_{f}\right)=\frac{T_{f}\left(x_{f}\right)-T_{\infty }}{T_{h1}-T_{c1}}, \end{array}$$

(10c) $$\begin{array}{c} m^{*}=2L_{f}\sqrt{\frac{h_{f}}{k_{f}d_{f}}}=\frac{2\sqrt{{\text{Bi}}_{f}}}{a_{f}}, \end{array}$$

where Bi_*f*_ = *h*_*f*_ *d*_*f*_/*k*_*f*_ is the pin Biot number and *a*_*f*_ = *d*_*f*_/*L*_*f*_ is the pin aspect ratio. The boundary conditions of (9) are given by

(11a) $$\begin{array}{c} \theta_{f}\left(X_{f}=0\right)=1-\theta_{h,W}-S, \end{array}$$

(11b) $$\begin{array}{c} \theta_{f}\left(X_{f}=1\right)=\theta_{c,W}-S, \end{array}$$

where *θ*_*h*,*W*_ = *θ*_*h*_(*X*_*h*_, *Y*_*h*_ = 1) and *θ*_*c*,*W*_ = *θ*_*c*_(*X*_*c*_ = 1 − *X*_*h*_, *Y*_*c*_ = 1) are the dimensionless temperatures of the hot and cold boundaries, respectively. *S* is the dimensionless cold excess temperature. It is equal to

(12) $$\begin{array}{c} S=\frac{T_{\infty }-T_{c1}}{T_{h1}-T_{c1}}, \end{array}$$

where 0 ≤ *S* ≤ 1 as auxiliary fluids are usually hotter than the cold reservoir. The solution of (9) is

(13) θf(Xf)=(1−θh,W−S)sinh⁡(m∗[1−Xf])sinh⁡(m∗) +(θc,W−S)sinh⁡(m∗Xf)sinh⁡(m∗).

Therefore, the conduction heat flux at the pin bases is equal to

(14) qcond′′|xf=0=−kfdTfdxf|xf=0=(1−θh,W−S)coth⁡(m∗)+(S−θc,W)csc h(m∗)Lf/[kfm∗(Th1−Tc1)],qcond′′|xf=Lf=−kfdTfdxf|xf=Lf=(1−θh,W−S)csc h(m∗)+(S−θc,W)coth⁡(m∗)Lf/[kfm∗(Th1−Tc1)].

Note that *q*_*fh*_′′ = *q*_cond_′′|_*x*_*f*_=0_ and *q*_*fc*_′′ = −*q*_cond_′′|_*x*_*f*_=*L*_*f*__. Recall that *q*_*uh*,*uc*_′′ = *h*_*u*_(*T*_*hW*,*cW*_ − *T*_*∞*_), where *T*_*hW*_ and *T*_*cW*_ are the temperatures at the hot and cold boundaries facing the auxiliary fluid, respectively. As such, (7c) and (7d) change to

(15a) ∂θh∂Yh|Yh=1={ϕ(kfkh)(HhLf)m∗coth⁡(m∗)+(1−ϕ)Bih} ×(1−θhW−S) +ϕ(kfkh)(HhLf)m∗csc h(m∗)(S−θcW),

(15b) ∂θc∂Yc|Yc=1={ϕ(kfkc)(HcLf)m∗coth⁡(m∗)+(1−ϕ)Bih(khkc)(HcHh)}(S−θcW) +ϕ(kfkc)(HcLf)m∗csc h(m∗)(1−θhW−S),

where Bi_*h*_ = *h*_*u*_ *H*_*h*_/*k*_*h*_ is the hot fluid Biot number.

### 2.3. The Heat Transfer Rates through the Heat Exchanger

The heat transfer rate per unit width from the hot fluid (*q*_*h*_′) and that to the cold fluid (*q*_*c*_′) can be calculated from

(16) qh′=ρh(cp)humhHh(Th1−Tmh2)=kh(Th1−Tc1)Pehθmh2,qc′=ρc(cp)cumcHc(Tmc2−Tc1)=kc(Th1−Tc1)Pecθmc2,

where *T*_*mh*2_ and *T*_*mc*2_ are the mean bulk temperatures at the hot and cold fluid exit ports, respectively. *θ*_*mh*2_ and *θ*_*mc*2_ are the dimensionless values of *T*_*mh*2_ and *T*_*mc*2_, respectively. In terms of the dimensionless parameters, *θ*_*mh*_ and *θ*_*mc*_ are given by

(17) $$\begin{array}{c} \theta_{mh,mc}=\overset{1}{\underset{0}{\int }}U_{h,c}\theta_{h,c}dY_{h,c}. \end{array}$$

The integral form of (2) can be expressed as

(18) $$\begin{array}{c} a_{h,c}\text{P}{\text{e}}_{h,c}\theta_{mh,mc}=\overset{1}{\underset{0}{\int }}{\frac{\partial \theta_{h,c}}{\partial Y_{h,c}}|}_{Y_{h,c}=1}dX_{h,c}. \end{array}$$

### 2.4. Modeling of the Pin Base Area Distribution over the Channel Boundary

**Figure 2:**
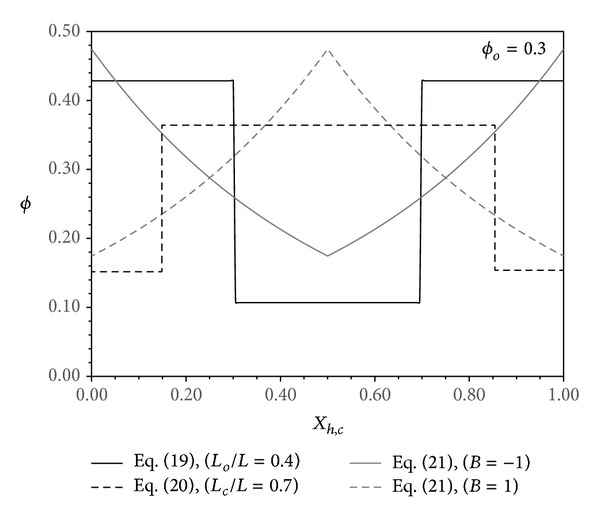
Variation of *ϕ* with *X*_*h*_ and *X*_*c*_ for the step distributions and exponential function.

Different distributions for pins will be analyzed in this work. These distributions allow having concentrated pin distribution either far from the middle section of the channels or about this section. Two families of distributions are considered. They are the step function and exponential distributions. The step function distribution that has concentrated pins far from the channels midsection (see Figure 2) has the following mathematical form:

(19) $$\begin{array}{c} \frac{\phi }{\phi_{o}}=[\begin{array}{cc} \frac{1-\left(1-\left[L_{o}/L_{m}\right]\right)\left(L_{o}/L\right)}{1-\left(L_{o}/L\right)}, & \\ \;\;0\leq 2X_{h,c}\leq 1-\left(\frac{L_{o}}{L}\right) & \\ 1-\left(\frac{L_{o}}{L_{m}}\right), & \\ \;\;1-\left(\frac{L_{o}}{L}\right)<2X_{h,c}<1+\left(\frac{L_{o}}{L}\right) & \\ \frac{1-\left(1-\left[L_{o}/L_{m}\right]\right)\left(L_{o}/L\right)}{1-\left(L_{o}/L\right)}, & \\ \;\;1+\left(\frac{L_{o}}{L}\right)\leq 2X_{h,c}\leq 2, & \end{array} \end{array}$$

where 0 ≤ (*L*_*o*_/*L*) ≤ (*L*_*m*_/*L*) = 1 − (*ϕ*_*o*_/*ϕ*_*m*_). *L*_*m*_ is the maximum value of *L*_*o*_ that produce 99% of the upper limit of pins base area concentration (*ϕ*_*m*_). *ϕ*_*m*_ when pins have parallel arrangement is equal to *ϕ*_*m*_ = 0.99(*π*/4). The quantity *ϕ*_*o*_ is the average pins base area concentration. To have the pins concentrated uniformly near channels midsection (see Figure 2), (19) changes to the following:

(20) $$\begin{array}{c} \frac{\phi }{\phi_{o}}=[\begin{array}{cc} \frac{\left(L_{c}/L\right)-\left(L_{m}/L\right)}{1-\left(L_{m}/L\right)}, & \\ \;\;0\leq 2X_{h,c}<1-\left(\frac{L_{c}}{L}\right) & \\ \frac{\left(L/L_{c}\right)+\left(L_{c}/L\right)\left[1-\left(L_{m}/L_{c}\right)\right]-1}{1-\left(L_{m}/L\right)}, & \\ \;\;1-\left(\frac{L_{c}}{L}\right)\leq 2X_{h,c}\leq 1+\left(\frac{L_{c}}{L}\right) & \\ \frac{\left(L_{c}/L\right)-\left(L_{m}/L\right)}{1-\left(L_{m}/L\right)}, & \\ \;\;1+\left(\frac{L_{c}}{L}\right)<2X_{h,c}\leq 2, & \end{array} \end{array}$$

where (*L*_*m*_/*L*) = (*ϕ*_*o*_/*ϕ*_*m*_) ≤ (*L*_*c*_/*L*) ≤ 1.

The exponential distribution of the pins has the following functional form:

(21) $$\begin{array}{c} \frac{\phi }{\phi_{o}}=\left\{\frac{B}{\exp\left(B\right)-1}\right\}\exp\left(B\left|1-2X_{h,c}\right|\right), \end{array}$$

where |*B*| < *B*_*c*_. *B*_*c*_ is the upper limit value that makes *ϕ* = *ϕ*_*m*_. It can be accurately correlated to *ϕ*_*o*_ through the following correlation:

(22) $$\begin{array}{c} B_{c}=\frac{-142-366\phi_{o}+699\phi_{o}^{2}}{1-209\phi_{o}-201\phi_{o}^{2}}. \end{array}$$

The pins are more concentrated near the channels inlet/exit sections when *B* > 0. However, they are more concentrated around the channels midsection when *B* < 0. These trends are seen in Figure 2.

### 2.5. The Heat Exchanger Performance Ratios

#### 2.5.1. Hot and Cold Fluid Flow Nusselt Numbers

The convection heat transfer coefficient for hot and cold fluid flows *h*_*h*_ and *h*_*c*_, respectively, is defined as

(23) $$\begin{array}{c} h_{h,c}\left\{T_{mh,mc}-T_{hW,cW}\right\}=-k_{h,c}{\frac{\partial T_{h,c}}{\partial y_{h,c}}|}_{y_{h,c}=H_{h,c}}. \end{array}$$

Thus, the local Nusselt numbers Nu_*h*_ and Nu_*c*_ are equal to

(24) $$\begin{array}{c} \text{N}{\text{u}}_{h,c}\equiv \frac{h_{h,c}H_{h,c}}{k_{h,c}}=\frac{-{\partial \theta_{h,c}/\partial Y_{h,c}|}_{Y_{h,c}=1}}{\theta_{mh,mc}-\theta_{hW,cW}}. \end{array}$$

#### 2.5.2. Heat Exchanger Effectiveness Ratios

The maximum heat transfer rate per unit width from the hot fluid *q*_*hMax*⁡_′ and that to the cold fluid *q*_*cMax*⁡_′ are obtainable when *T*_*h*2_ = *T*_*c*1_ and *T*_*c*2_ = *T*_*h*1_. Using (16), *q*_*hMax*⁡_′ and *q*_*cMax*⁡_′ are equal to

(25) $$\begin{array}{c} \left(q_{h\mathrm{Max}}^{\prime },q_{c\mathrm{Max}}^{\prime }\right)=\left(k_{h},k_{c}\right)\left(T_{h1}-T_{c1}\right)\left({\text{Pe}}_{h},{\text{Pe}}_{c}\right). \end{array}$$

Define the heat exchanger effectiveness factor *ε*_*h*_ as the ratio of heat transfer rate from the hot fluid to *q*_*hMax*⁡_. Also, *ε*_*c*_ is defined as the ratio of heat transfer rate to the cold fluid to *q*_*cMax*⁡_. As such, *ε*_*h*_ and *ε*_*c*_ are mathematically equal to

(26) $$\begin{array}{c} \left(\varepsilon_{h},\varepsilon_{c}\right)\equiv \frac{\left(q_{h}^{\prime },q_{c}^{\prime }\right)}{\left(q_{h\mathrm{Max}}^{\prime },q_{c\mathrm{Max}}^{\prime }\right)}=\left(\theta_{mh2},\theta_{mc2}\right). \end{array}$$

#### 2.5.3. Heat Exchanger Second Set of Performance Ratios

Let the reference case for the second performance ratios be the counterflow heat exchanger with perfect indirect contact between the hot and cold fluids. For this case, the boundary conditions given by (7c) and (7d) change to

(27a) $$\begin{array}{c} {\frac{\partial \theta_{h}}{\partial Y_{h}}|}_{Y_{h}=1}=\left(\frac{H_{h}}{H_{c}}\right)\left(\frac{k_{c}}{k_{h}}\right)\left({\frac{\partial \theta_{c}}{\partial Y_{c}}|}_{Y_{c}=1}\right), \end{array}$$

(27b) $$\begin{array}{c} \theta_{c,W}=1-\theta_{h,W}. \end{array}$$

The heat transfer rate between the two fluids for this case, *q*_*o*_′, is equal to

(28) qo′=kh(Th1−Tc1)Peh[θmh2]o=kc(Th1−Tc1)Peh[θmc2]o,

where [*θ*_*mh*2_]_*o*_ and [*θ*_*mc*2_]_*o*_ are the dimensionless exit mean bulk temperatures of hot and cold fluids, respectively, for the reference case. Define the heat exchanger performance indicators *γ*_*h*_ and *γ*_*c*_ as the ratio of heat transfer rate from the hot fluid and that to the cold fluid, respectively, to the reference heat transfer rate. Mathematically, they are equal to

(29) $$\begin{array}{c} \left(\gamma_{h},\gamma_{c}\right)\equiv \frac{\left(q_{h}^{\prime },q_{c}^{\prime }\right)}{q_{o}^{\prime }}=\frac{\left(\theta_{mh2},\theta_{mc2}\right)}{\left({\left[\theta_{mh2}\right]}_{o},{\left[\theta_{mc2}\right]}_{o}\right)}. \end{array}$$

#### 2.5.4. Heat Exchanger Set of Performance Ratios due to Stratified Pin Distribution

The last set of performance indicators for the present heat exchanger denoted by (*λ*_*h*_, *λ*_*c*_) are defined as the ratios of the heat transfer rate from the hot fluid and that to the cold fluid to the corresponding quantities when *ϕ* = *ϕ*_*o*_, respectively. Mathematically, they are equal to

(30) $$\begin{array}{c} \left(\lambda_{h},\lambda_{c}\right)\equiv \frac{\left(q_{h}^{\prime },q_{c}^{\prime }\right)}{{\left(q_{h}^{\prime },q_{c}^{\prime }\right)|}_{\phi =\phi_{o}}}=\frac{\left(\theta_{mh2},\theta_{mc2}\right)}{{\left(\theta_{mh2},\theta_{mc2}\right)|}_{\phi =\phi_{o}}}. \end{array}$$

### 2.6. Analytical Model

Utilizing (15a), (15b), and (24), it can be shown that

(31) ThW−T∞=B1Nuh(Tmh−T∞)+B2(Hh/Hc)(kc/kh)Nuc(Tmc−T∞)B12+B22,TcW−T∞=B2Nuh(Tmh−T∞)+B1(Hh/Hc)(kc/kh)Nuc(Tmc−T∞)B12+B22,

where *h*_*h*,*c*_ ≪ *h*_*u*_. This condition is necessary as the aim of introducing the auxiliary fluid flow is to enhance heat transfer inside the hot and cold fluid channels. The coefficients *B*_1_ and *B*_2_ are given by

(32) $$\begin{array}{c} B_{1}=\phi \left(\frac{k_{f}}{k_{h}}\right)\left(\frac{H_{h}}{L_{f}}\right)m^{*}\coth\left(m^{*}\right)+\left(1-\phi \right)\text{B}{\text{i}}_{h},\\ B_{2}=\phi \left(\frac{k_{f}}{k_{h}}\right)\left(\frac{H_{h}}{L_{f}}\right)m^{*}\text{csc}\;h\left(m^{*}\right). \end{array}$$

Using (31), the heat transfer rates at the hot and cold differential boundary elements are given by

(33) δqh,c′=−kh,cPeh,cdTmh,mc=hh,c(Tmh,mc−ThW,cW)dxh,c=hh,cβ(Tmh,mc−T∞)dxh,c,

where *β* is equal to

(34) $$\begin{array}{c} \beta =\frac{B_{1}^{2}-B_{2}^{2}}{B_{1}^{2}+B_{2}^{2}}. \end{array}$$

Both sides of (33) can be arranged by separation of variables to the following differential equation:

(35) $$\begin{array}{c} \frac{dT_{mh,mc}}{T_{mh,mc}-T_{\infty }}=-\left[\frac{\beta \text{N}{\text{u}}_{h,c}}{a_{h,c}\text{P}{\text{e}}_{h,c}}\right]dX_{h,c}. \end{array}$$

Integrating (35) over the heat exchanger length results in the following solution:

(36) $$\begin{array}{c} \frac{T_{mh2,mc2}-T_{\infty }}{T_{mh1,mc1}-T_{\infty }}=\exp\left(-\frac{\int_{0}^{1}\beta \text{N}{\text{u}}_{h,c}dX_{h,c}}{a_{h,c}\text{P}{\text{e}}_{h,c}}\right). \end{array}$$

Using (26), *ε*_*h*_ and *ε*_*c*_ can be shown to be equal to

(37) $$\begin{array}{c} \varepsilon_{h}=\left(1-S\right)\left\{1-\exp\left(-\frac{\int_{0}^{1}\beta \text{N}{\text{u}}_{h}dX_{h}}{a_{h}\text{P}{\text{e}}_{h}}\right)\right\},\\ \varepsilon_{c}=S\left\{1-\exp\left(-\frac{\int_{0}^{1}\beta \text{N}{\text{u}}_{c}dX_{c}}{a_{c}\text{P}{\text{e}}_{c}}\right)\right\}. \end{array}$$

#### 2.6.1. Approximate Nusselt Number Correlation

Approximate Nusselt numbers {Nu_h_*, Nu_c_*} can be obtained when assuming that the pins are very long such that the heat transfer rates in both channels are uncoupled. For this case, (15a) and (15b) reduce to

(38a) $$\begin{array}{c} {\frac{\partial \theta_{h}}{\partial Y_{h}}|}_{Y_{h}=1}={\left({\text{Bi}}_{h}\right)}_{\text{eff}}\left(1-\theta_{h,W}-S\right), \end{array}$$

(38b) $$\begin{array}{c} {\frac{\partial \theta_{c}}{\partial Y_{c}}|}_{Y_{c}=1}={\left({\text{Bi}}_{c}\right)}_{\text{eff}}\left(S-\theta_{c,W}\right), \end{array}$$

where (Bi_*h*_)_eff_ and (Bi_*c*_)_eff_ are equal to

(39) $$\begin{array}{c} {\left(\text{B}{\text{i}}_{h}\right)}_{\text{eff}}=\phi \left(\frac{k_{f}}{k_{h}}\right)\left(\frac{H_{h}}{L_{f}}\right)m^{*}+\left(1-\phi \right)\text{B}{\text{i}}_{h},\\ {\left(\text{B}{\text{i}}_{c}\right)}_{\text{eff}}=\phi \left(\frac{k_{f}}{k_{c}}\right)\left(\frac{H_{c}}{L_{f}}\right)m^{*}+\left(1-\phi \right)\text{B}{\text{i}}_{h}\left(\frac{k_{h}}{k_{c}}\right)\left(\frac{H_{c}}{H_{h}}\right). \end{array}$$

The solutions of (2) for this case under fully developed flow condition can be numerically obtained with high accuracy by following the methodology described in Section 3 of this work. For this case, it can be shown that the following correlations of Nusselt numbers

(40) {Nuh,Nuc}≅{Nuh∗,Nuc∗}=2.695+0.9897{(Bih)eff,(Bic)eff}1+0.4076{(Bih)eff,(Bic)eff}

produce maximum error (relative) less than 0.1% when 0.02 ≤ {(Bi_*h*_)_eff_, (Bi_*c*_)_eff_} ≤ 100. It can be noticed form (40) that the Nusselt numbers are not strongly dependent on {(Bi_*h*_)_eff_, (Bi_*c*_)_eff_} values as the maximum variation (relative) in the Nusselt numbers is less than 10%.

#### 2.6.2. Upper Limits of Heat Fluxes due to Only Pin Conduction at Fully Developed Conditions

The maximum heat fluxes due to only pin conduction are obtained when (*T*_*mh*_, *T*_*mc*_) = (*T*_*h*1_, *T*_*c*1_) and Bi_*h*_ → 0. Accordingly, their dimensionless quantities are

(41) Θfh=ϕ(qfh′′)Max⁡(kh/Hh)(Th1−Tc1)=(1−S)×{2.695+1.9794ϕZhBif1+0.8152ϕZhBif} ×{sinh⁡2(2Bif/af)cosh⁡2(2Bif/af)+1},Θfc=−ϕ(qfc′′)Max⁡(kh/Hh)(Th1−Tc1)=S×{Hh/Hckh/kc}{2.695+1.9794ϕZcBif1+0.8152ϕZcBif} ×{sinh⁡2(2Bif/af)cosh⁡2(2Bif/af)+1},

where *Z*_*h*_ = (*k*_*f*_/*k*_*h*_)(*H*_*h*_/*d*_*f*_) and *Z*_*c*_ = (*k*_*f*_/*k*_*c*_)(*H*_*c*_/*d*_*f*_).

#### 2.6.3. Upper Limits of Heat Fluxes in Absence of Pins at Fully Developed Condition

The dimensionless maximum convection heat fluxes in absence of pins can be obtained when (*T*_*mh*_, *T*_*mc*_) = (*T*_*h*1_, *T*_*c*1_) and *k*_*f*_ → 0. They are equal to

(42) Θuh=(1−ϕ)(quh′′)Max⁡(kh/Hh)(Th1−Tc1)=(1−S)×(1−ϕ){2.695+0.9897Bih1+0.4076Bih},Θuc=−(1−ϕ)(quc′′)Max⁡(kh/Hh)(Th1−Tc1)=S×(1−ϕ){Hh/Hckh/kc}{2.695+0.9897Bic1+0.4076Bic},

where Bi_*c*_ = Bi_*h*_(*k*_*h*_/*k*_*c*_)(*H*_*c*_/*H*_*h*_).

### 2.7. Modeling of Auxiliary Fluid Flow Convection Coefficients and Minimum *ϕ*_*o*_

Let the auxiliary stream be laminar flow along the direction of the channels width axis. Therefore, the convection heat transfer coefficients for the fin and unfinned surfaces can be computed using the following correlations [19]:

(43) Nudf=hfdfke=0.3+0.62Redf1/2Pre1/3[1+(0.4/Pre)2/3]1/4×[1+(Redf282,000)5/8]4/5,  RedfPre>0.2,NuW=huWke=0.664ReW1/2Pre1/3,ReW<5  ×  105;  Pre>0,

where *k*_*e*_, Pr_*e*_, and *ν*_*e*_ are the auxiliary fluid thermal conductivity, Prandtl number, and kinematic viscosity, respectively. *u*_*∞*_ is the auxiliary free velocity. *Re*_*d*_*f*__ = *u*_*∞*_ *d*_*f*_/*ν*_*e*_ and *Re*_*W*_ = *u*_*∞*_ *W*/*ν*_*e*_ are the Reynolds numbers for the streams across the pins and along the unfinned surface, respectively. Thus, Bi_*h*_, m* and the relationships between the latter Reynolds numbers are equal to

(44) $$\begin{array}{c} \text{B}{\text{i}}_{h}=\left(\frac{k_{e}}{k_{h}}\right)\left(\frac{H_{h}}{W}\right)\text{N}{\text{u}}_{W};\;\;m^{*}=2\frac{\sqrt{\left(k_{e}/k_{f}\right)\text{N}{\text{u}}_{d_{f}}}}{a_{f}},\\ \text{R}{\text{e}}_{d_{f}}=\left(\frac{d_{f}}{W}\right)\text{R}{\text{e}}_{W}. \end{array}$$

The minimum requirements for average pin base area concentration can be obtained when the heat transfer rates between (hot, cold) fluids and auxiliary stream are equal to those between the hot and cold fluids under perfect indirect contact between the fluids. These quantities can be obtained analytically for the ideal cases: (1)  *a*_*h*_Pe_*h*_ and *k*_*f*_ are very large, while *a*_*c*_Pe_*c*_ is very small and (2)  *a*_*c*_Pe_*c*_ and *k*_*f*_ are very large, while *a*_*h*_Pe_*h*_ is very small. For these conditions, the minimum average pin base area concentration denoted by *ϕ*_*hMin*⁡,*cMin*⁡_, respectively, can be shown to be equal to

(45) $$\begin{array}{c} \phi_{h\mathrm{Min}}=\frac{\left(k_{c}/k_{e}\right)\left(W/H_{c}\right)a_{c}\text{P}{\text{e}}_{c}/\left[\left(1-S\right)\text{N}{\text{u}}_{W}\right]-1}{\left(4/a_{f}\right)\left(W/d_{f}\right)\text{N}{\text{u}}_{d_{f}}/\text{N}{\text{u}}_{W}-1},\\ \phi_{c\mathrm{Min}}=\frac{\left(k_{h}/k_{e}\right)\left(W/H_{h}\right)a_{h}\text{P}{\text{e}}_{h}/\left[S\text{N}{\text{u}}_{W}\right]-1}{\left(4/a_{f}\right)\left(W/d_{f}\right)\text{N}{\text{u}}_{d_{f}}/\text{N}{\text{u}}_{W}-1}. \end{array}$$

## 3. Numerical Methodology and Results

Equation (2) is coupled via the boundary conditions given by (15a) and (15b). These equations can be solved by iterations using the implicit finite difference method discussed by Khaled and Vafai [23]. Equation (2) was discretized by employing three-point central differencing quotients for the first and second derivatives with respect to *Y*_*h*,*c*_ directions. Furthermore, two-point backward differencing quotients were used in the discretization of the first derivatives with respect to *X*_*h*,*c*_ directions. For (15a) and (15b), three-point central differencing quotients were used to discretize the first derivatives with respect to *Y*_*h*,*c*_ directions. The finite difference equations of (2) are given by

(46) ahPeh(Uh)j{(θh)i,j−(θh)i−1,jΔXh}  ={(θh)i,j−1−2(θh)i,j+(θh)i,j+1ΔYh2},acPec(Uc)J{(θc)I,J−(θc)I−1,JΔXc}  ={(θc)I,J−1−2(θc)I,J+(θc)I,J+1ΔYc2}.

The pairs (*i*, *j*) and (*I* = *M* − *i* + 1, *J*) represent the location of the discretized points in the numerical grids of the hot and cold fluid domains, respectively. *M* is the total number of either *i* or *I* sections. *N*_*h*_ and *N*_*c*_ are the total number of the discretized points per *i* and *I* sections, respectively. *M*, *N*_*h*_, and *N*_*c*_ were taken to be equal to *M* = 1001 and *N*_*h*_ = *N*_*c*_ = 201.

The applications of (46) for all discretized points at given *i* and *I* sections result in *N*_*h*_ and *N*_*c*_ tridiagonal systems of algebraic equations. These equations can easily be solved using the Thomas algorithm [24], if internal boundary temperatures are known. The numerical solution procedure is summarized in the following steps.

(1) The dimensionless cold boundary temperatures were assumed (*θ*_*cW*_)_*i*_^assumed^.
(2) The *N*_*h*_ algebraic equations for *i* = 2,…, *M* were solved.
(3) The *N*_*c*_ algebraic equations for *I* = 2,…, *M* were solved.
(4) The corrected cold boundary temperatures (*θ*_*cW*_)_*i*_^corrected^ were found using the finite difference equation of (15b).
(5) Steps (2)–(4) were repeated by replacing (*θ*_*cW*_)_*i*_^assumed^ with the corrected (*θ*_*cW*_)_*i*_^corrected^ until the following condition is satisfied:
  (47) $$\begin{array}{c} \mathrm{Max}\left|\frac{{\left(\theta_{cW}\right)}_{i}^{\text{corrected}}-{\left(\theta_{cW}\right)}_{i}^{\text{corrected}}}{{\left(\theta_{cW}\right)}_{i}^{\text{corrected}}}\right|<10^{-5}. \end{array}$$

**Figure 3:**
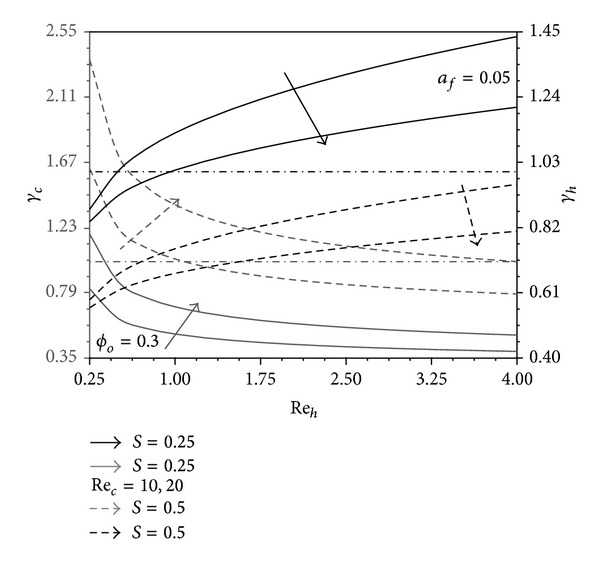
Effects of Re_*h*_ and Re_*c*_ on *γ*_*h*_ and *γ*_*c*_.

**Figure 4:**
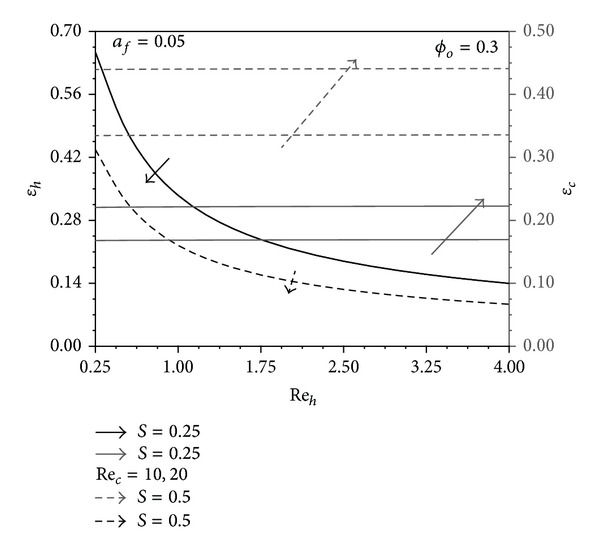
Effects of Re_*h*_ and Re_*c*_ on *ε*_*h*_ and *ε*_*c*_.

**Figure 5:**
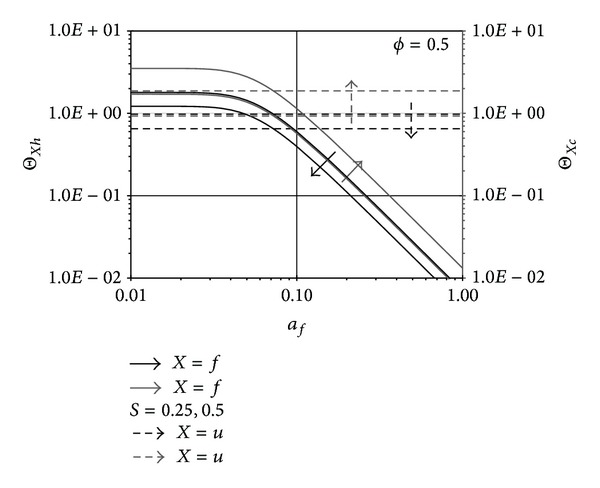
Effects of *a*_*f*_ on (Θ_*fh*_, Θ_*fc*_) and (Θ_*uh*_, Θ_*uc*_).

**Figure 6:**
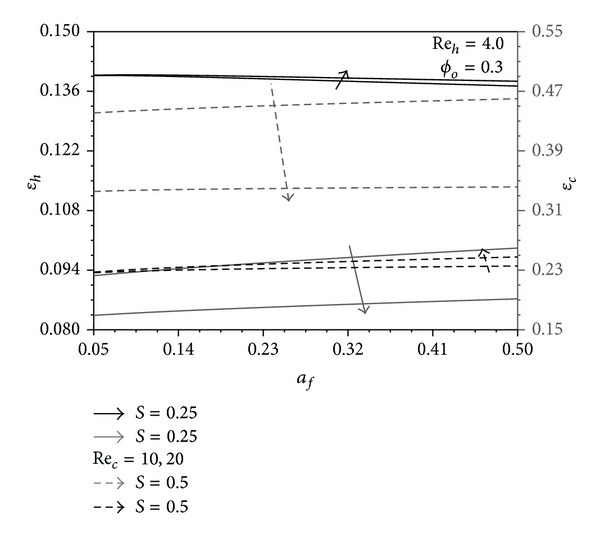
Effects of *a*_*f*_ and Re_*c*_ on *ε*_*h*_ and *ε*_*c*_.

**Figure 7:**
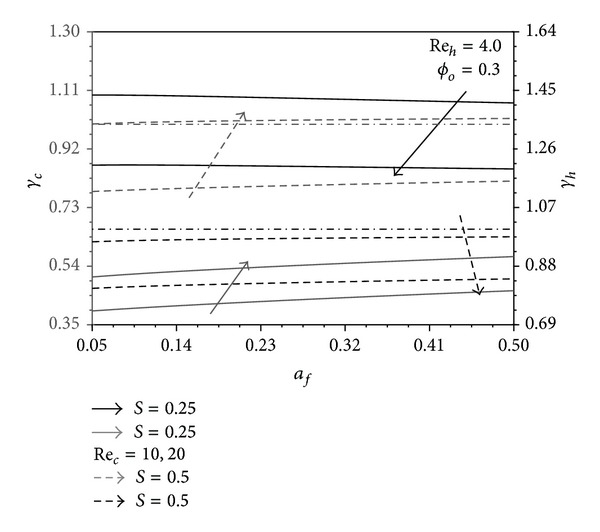
Effects of *a*_*f*_ and Re_*c*_ on *γ*_*h*_ and *γ*_*c*_.

**Figure 8:**
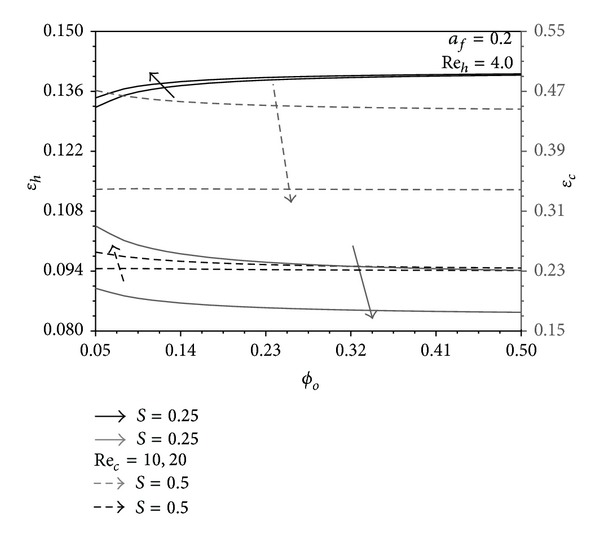
Effects of *ϕ*_*o*_ and Re_*c*_ on *ε*_*h*_ and *ε*_*c*_.

**Figure 9:**
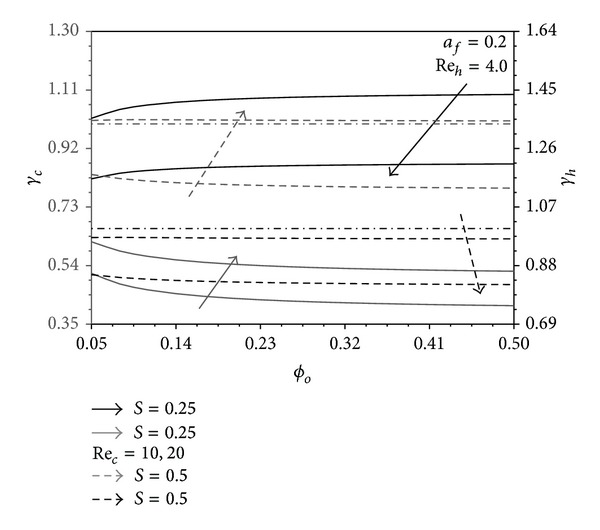
Effects of *ϕ*_*o*_ and Re_*c*_ on *γ*_*h*_ and *γ*_*c*_.

**Figure 10:**
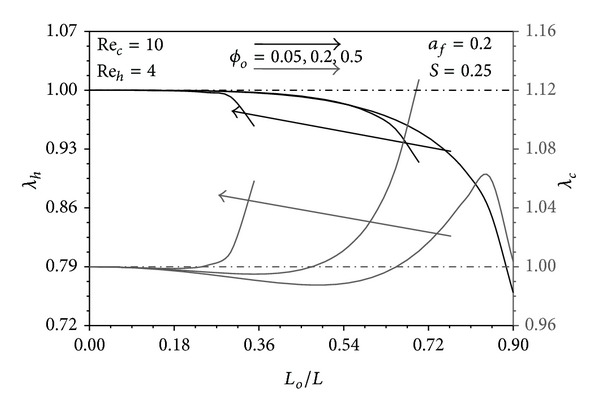
Effects of *L*_*o*_/*L* and *ϕ*_*o*_ on *λ*_*h*_ and *λ*_*c*_.

**Figure 11:**
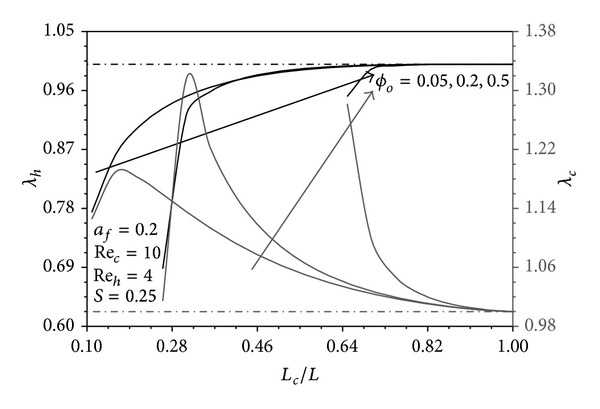
Effects of *L*_*c*_/*L* and *ϕ*_*o*_ on *λ*_*h*_ and *λ*_*c*_.

**Figure 12:**
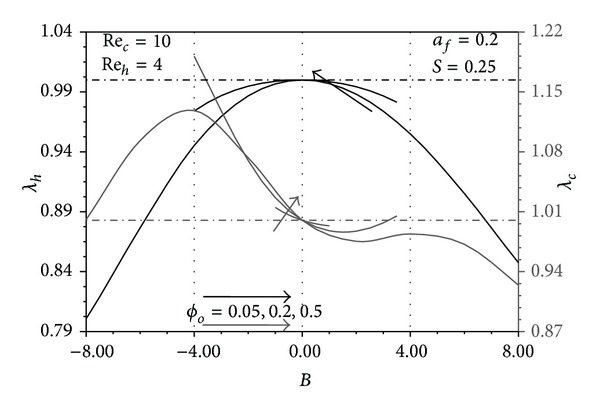
Effects of *B* and *ϕ*_*o*_ on *λ*_*h*_ and *λ*_*c*_.

Using doubled mesh sizes results in less than 0.3% error in the calculated parameters for moderate *a*_*h*_Pe_*h*_ and *a*_*c*_Pe_*c*_ values. This ascertains the grid-size independent results. The numerical results shown in Figures 3–12 were generated for the hot, cold, and the auxiliary stream fluids shown in Table 1. These selections correspond to an important application which is oil cooling cold water stream augmented by an air stream. The numerical results were compared with the analytical solution given by (35), (36), and (40) as shown in Table 2. The numerical results are seen to have a good agreement with the analytical solution as *a*_*h*,*c*_Pe_*h*,*c*_ ≪ 1. The latter constraint is the major assumption used to generate (40).

## 4. Discussion of the Results

### 4.1. The Role of Internal Flow Reynolds Numbers in the Performance Ratios

As Re_*h*_ increases, both hot fluid mean bulk and heated boundary temperatures decrease due to the increase in advection and the widening effect of the thermal entry region. As a result, the convection heat transfer rate to the auxiliary stream and conduction through the pins increase due to the increase in the heated boundary and pins excess temperatures (*T*_*hW*_ − *T*_*∞*_, *T*_*hW*_ − *T*_*cW*_), respectively. These excess temperatures increase as *S* decreases. Accordingly, the heat transfer rate from the hot fluid increases which causes *γ*_*h*_ to increase as both Re_*h*_ and (1 − *S*) increase as seen in Figure 3. This figure shows that *γ*_*h*_ decreases as Re_*c*_ increases. This indicates that the increase in the reference case heat transfer rate (*q*_*o*_′) is larger than that for the present system (*q*_*h*_′). Also, it is noticed from Figure 3 that *γ*_*h*_ can be larger than one at both smaller *S* and Re_*c*_ values and larger Re_*h*_ values. Similarly, *γ*_*c*_ can be larger than one for smaller (1 − *S*) and Re_*h*_ values and larger Re_*c*_ values.

The increase in Re_*h*_ decreases the hot fluid effectiveness *ε*_*h*_ as shown in Figure 4, since it majorly results in reduction of the hot fluid mean bulk temperature. However, a decrease in *S* is noticed to cause an increase in *ε*_*h*_ due to the associated increase in *T*_*hW*_ − *T*_*∞*_. Figures 7 and 9 show that an increase in Re_*c*_ causes an increase in *ε*_*h*_. This is because both *T*_*∞*_ − *T*_*cW*_, *T*_*hW*_ − *T*_*cW*_ increase as Re_*c*_ increases; thus, the conduction through the pins is enhanced. The latter enhancement cannot be clearly identified from Figure 4 as the pin aspect ratio is very small for this figure which is *a*_*f*_ = 0.05. As indicated earlier, when *a*_*f*_ → 0 the heat transfer rate in both channels will be uncoupled. In a similar manner, it can be concluded that *ε*_*c*_ decreases as Re_*c*_ increases and as (1 − *S*) decreases and it increases as Re_*h*_ increases as noticed in Figure 4.

### 4.2. The Role of Pins Aspect Ratio in the Performance Ratios

As *L*_*f*_ increases (*a*_*f*_ decreases), the pin surface area increases causing the fully developed maximum fluxes Θ_*fh*_ and Θ_*fc*_ to increase until they reach their asymptotic values as shown in Figure 5. This causes *ε*_*h*_ to increase as *a*_*f*_ decreases when *S* = 0.25 as shown in Figure 6. When *S* = 0.5, Θ_*fc*_ ≫ Θ_*fh*_; thus, *T*_*cW*_ sharply increases causing sharp reduction in *T*_*hW*_ − *T*_*cW*_. Therefore, *ε*_*h*_ decreases as *a*_*f*_ decreases when *S* = 0.5. The cases considered in Figures 6 and 7 have *a*_*h*_Pe_*h*_ ≫ *a*_*c*_Pe_*c*_ > 1. Thus, the hot fluid flow is dominated by the thermal entry region. For this condition, *T*_*hW*_ is less sensitive to *L*_*f*_, while *T*_*cW*_ increases apparently as *L*_*f*_ increases. As a result, *T*_*∞*_ − *T*_*cW*_,  and *T*_*hW*_ − *T*_*cW*_ decrease as *L*_*f*_ increases. Since pins conduction is linearly dependent on *T*_*∞*_ − *T*_*cW*_, and *T*_*hW*_ − *T*_*cW*_ while it is less sensitive to *L*_*f*_, *ε*_*c*_ decreases as *a*_*f*_ decreases as seen in Figure 6. Due to the previous analysis, *γ*_*h*_ and *γ*_*c*_ increase as *a*_*f*_ increases except when *S* = 0.25 where *γ*_*h*_ is noticed in Figure 7 to decrease as *a*_*f*_ increases. Also, it is shown from this figure that *γ*_*h*_ > 1 when *S* = 0.25 and Re_*c*_ = 10 as well as *γ*_*c*_ > 1 when *S* = 0.5 and Re_*c*_ = 20. This demonstrates the superiority of the present heat exchanger over the conventional counterflow plate heat exchanger.

### 4.3. The Role of Pins Base Area Concentration in the Performance Indicators

Two limiting cases can be encountered in the present heat exchanger. They are as follows: (1) pure convection between the channels and the auxiliary stream when *ϕ*_*o*_ → 0 and (2) pure conduction between the channels when *ϕ*_*o*_ → *ϕ*_*m*_. As seen in Figure 5, (Θ_*uh*_, Θ_*uc*_) > (Θ_*fh*_, Θ_*fc*_) when *a*_*f*_ = 0.2 which represents the condition for Figures 8 and 9. Accordingly, (*ε*_*h*_, *ε*_*c*_) pair is expected to decrease as *ϕ*_*o*_ increases. This is shown in Figure 8 except for the *ε*_*h*_ plot when *S* = 0.25. For this case, *T*_*hW*_ − *T*_*∞*_, and *T*_*hW*_ − *T*_*cW*_ are very close to their upper limit (*T*_*h*1_ − *T*_*c*1_) while this limit is reduced to (*T*_*h*1_ − *T*_*∞*_) for the pure convection condition as *ϕ*_*o*_ → 0. And since the heat flux is linearly proportional *T*_*hW*_ − *T*_*∞*_, and *T*_*hW*_ − *T*_*cW*_ as can be seen in (15a) and (15b), *ε*_*h*_ is increased when *ϕ*_*o*_ is increased for *S* = 0.25. Because of the previous facts, (*γ*_*h*_, *γ*_*c*_) pair decreases as *ϕ*_*o*_ increases except when *S* = 0.25 where *γ*_*h*_ is noticed in Figure 9 to increase as *ϕ*_*o*_ increases. Furthermore, it is shown from this figure that *γ*_*h*_ > 1 when *S* = 0.25 and Re_*c*_ = 10 while *γ*_*c*_ > 1 when *S* = 0.5. This demonstrates the superiority of the present heat exchanger over the conventional counterflow plate heat exchanger.

### 4.4. The Role of Pins Distribution in the Performance Indicators

**Figure 13:**
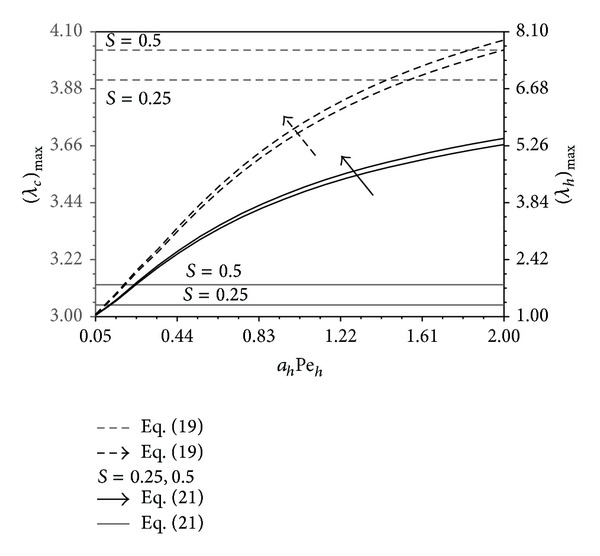
Effects of *a*_*h*_Pe_*h*_ and *S* on (*λ*_*h*_)_max⁡_ and (*λ*_*c*_)_max⁡_ (0.01 ≤ *a*_*f*_ ≤ 0.25, 0.12 ≤ *a*_*c*_Pe_*c*_ ≤ 0.6, 0.05 ≤ *ϕ*_*o*_ ≤ 0.5) (−*B*_*c*_ ≤ *B* ≤ 0.6 ≤ *B*_*c*_  (or)  0 ≤ *L*_*o*_/*L* ≤ *L*_*m*_/*L*).

**Figure 14:**
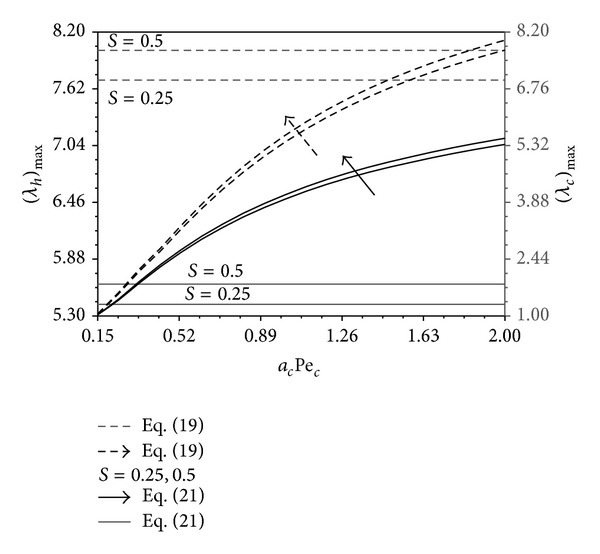
Effects of *a*_*c*_Pe_*c*_ and *S* on (*λ*_*c*_)_max⁡_ and (*λ*_*h*_)_max⁡_ (0.01 ≤ *a*_*f*_ ≤ 0.25, 0.05 ≤ *a*_*h*_Pe_*h*_ ≤ 2, 0.05 ≤ *ϕ*_*o*_ ≤ 0.5) (−*B*_*c*_ ≤ *B* ≤ 0.6 ≤ *B*_*c*_  (or)  0 ≤ *L*_*o*_/*L* ≤ *L*_*m*_/*L*).

Since *S* = 0.25 and *a*_*h*_Pe_*h*_ ≫ *a*_*c*_Pe_*c*_ > 1 in Figures 10, 11, and 12, *T*_*hW*_ − *T*_*∞*_, and *T*_*hW*_ − *T*_*cW*_ are very close to the maximum value (*T*_*h*1_ − *T*_*c*1_). These excess temperatures become closer to that limit near the hot fluid inlet. Far from this region, they tend to apparently decrease as *ϕ* decreases since Θ_*uh*_ > Θ_*fh*_. Consequently, the uniform distribution of pins reveals the maximum *λ*_*h*_ in which *λ*_*h*_ = 1 as seen from these figures. On the other hand, *T*_*∞*_ − *T*_*cW*_ turns out to be much smaller than (*T*_*∞*_ − *T*_*c*1_) particularly near the cold fluid exit and as *ϕ* decreases because Θ_*uc*_ > Θ_*fc*_. But the pin conduction from hot fluid to the cold fluid increases as *ϕ* decreases near the cold/hot fluid exit/inlet as explained earlier. Accordingly, *λ*_*c*_ is maximized for specific *ϕ* distribution distinguished by having critical values of *L*_*o*_/*L*, *L*_*c*_/*L* and B as shown in Figures 10–12. Increases in *a*_*h*_Pe_*h*_, and *a*_*c*_Pe_*c*_ reduce *T*_*mh*_, and *T*_*mc*_, respectively. As a result, *T*_*hW*_ − *T*_*∞*_, and *T*_*∞*_ − *T*_*cW*_ increase causing {(*λ*_*h*_)_max⁡_, (*λ*_*c*_)_max⁡_} to increase as *a*_*h*_Pe_*h*_, and *a*_*c*_Pe_*c*_ increase, respectively. These trends are seen clearly in Figures 13 and 14. These maximum ratios are obtained based on {Nu_h_*, Nu_c_*} approximations. Some critical parameters that produce the maximum quantities {(*λ*_*h*_)_max⁡_, (*λ*_*c*_)_max⁡_} are listed in Table 3. Finally, {(*λ*_*h*_)_max⁡_, (*λ*_*c*_)_max⁡_} as seen from Figures 13 and 14 can be much larger than the one indicating that properly distributing the pins is an efficient mechanism for heat transfer enhancement under fully developed laminar flow condition.

## 5. Conclusions

Heat transfer inside counter-flow plate heat exchanger subject to internal convections with an auxiliary fluid was investigated in this work. The auxiliary fluid passage is surrounded by the hot and cold fluid channels and it is supported by highly conductive pins connected to both channels. Good agreement was noticed between the numerical solution and an approximate analytical solution based on fully developed flow and very long pin conditions. The results of the current study can be summarized by the following concluding remarks.

1. The heat transfer rate from/to the hot/cold fluid of the present system can be higher than that for the conventional counterflow plate heat exchanger under the following conditions: (1) large hot/cold flow Reynolds number, (2) small cold/hot flow Reynolds number, and (3) large hot/cold fluid excess temperature.
2. Increasing either the number pins or pins length may increase the hot/cold heat transfer rate above that for the conventional counterflow plate heat exchanger under the following conditions: (1) effective hot/cold fluid Biot number due to only pins conduction being larger than that due to only convection with the unfinned surface and (2) large hot/cold fluid excess temperature as when having large hot/cold flow Reynolds number.
3. Uniform distribution of the pins leads to maximum heat transfer enhancement ratios when thermal entry regions are significant.
4. Larger heat transfer enhancement ratios are obtained when both hot and cold fluid flows are fully developed.
5. The maximum enhancement factors can be increased by above 8.0- and 5.0-fold for the step and exponential distributions of the pins, respectively.

Finally, the counterflow plate heat exchanger with auxiliary intermediate channel can be recommended over the conventional one, if the auxiliary fluid flow is provided with negligible cost.

## Figures and Tables

**Table 1:** The used fixed dimensions and the properties of different substances.

Property	Hot fluid	Cold fluid	Auxiliary fluid	Auxiliary fluid
	Engine oil	Water	Air	Air
*T*_1_ (°C)	80	15	31.25 (*S* = 0.25)	47.5 (*S* = 0.5)
*c*_*p*_ (*J* kg^−1^ K^−1^)	2132	4186	1007	1008
*k* (*W* m^−1^ K^−1^)	0.138	0.595	0.0266	0.0278
*μ* (kg m^−1^ s^−1^)	0.0323	1.134 × 10^−3^	1.867 × 10^−5^	1.943 × 10^−5^
*ρ* (kg m^−3^)	852	999	1.228	1.101
Dimension	Hot flow channel	Cold flow channel	Dimension and property	Pins
*H* (mm)	2	3	*d*_*f*_ (mm)	2
*W* (mm)	48	48	*k*_*f*_ (*W* m^−1^ K^−1^)	401
*L* (mm)	200	200	*u*_*∞*_ (m s^−1^)	25
(*S* = 0.25)	${\left(\phi_{h,c}\right)}_{\mathrm{Min}}=\frac{\left(0.04308,0.02997\right){\text{Pe}}_{c,h}-1}{17.028/a_{f}-1}$			
(*S* = 0.5)	${\left(\phi_{h,c}\right)}_{\mathrm{Min}}=\frac{\left(0.06668,0.01547\right){\text{Pe}}_{c,h}-1}{16.966/a_{f}-1}$			

**Table 2:** A comparison between the numerical solution and the analytical one given by (37) and (40).

Quantity	*a*_*c*_Pe_*c*_ = 0.5954, ( Re_*c*_ = 5)			
	Re_*h*_ = 0.01	Re_*h*_ = 0.05	Re_*h*_ = 0.09	Re_*h*_ = 0.011
*a*_*h*_Pe_*h*_	0.05	0.25	0.45	0.55
*ε*_*h*_ (Analytical)	0.75	0.7497	0.7405	0.7290
*ε*_*h*_ (Numerical)	0.7526	0.7515	0.7480	0.7427
*ε*_*c*_ (Analytical)	0.2408			
*ε*_*c*_ (Numerical)	0.2460	0.2465	0.2468	0.2469

**Table 3:** Critical parameters for *λ*_*h*,max⁡_ (*) and *λ*_*c*,max⁡_ (**) as functions of *a*_*h*_Pe_*h*_ and *a*_*c*_Pe_*c*_, respectively (0.01 ≤ *a*_*f*_ ≤ 0.25,0.12 ≤ *a*_*c*_Pe_*c*_ ≤ 0.6,0.5 ≤ *a*_*h*_Pe_*h*_ ≤ 2.14,0.05 ≤ *ϕ*_*o*_ ≤ 0.5).

*a*_*h*_Pe_*h*_	*S*	*a*_*f*_	*a*_*c*_Pe_*c*_	Case with (19)		Case with (21)	
				*L*_*o*_/*L*	*ϕ*_*o*_	*B*	*ϕ*_*o*_
0.05	0.25	0.25	0.12	0.4	0.466	−5.342	0.146
0.05	0.5	0.25	0.12	0.4	0.466	−5.342	0.146
2.05	0.25	0.25	0.12	0.75	0.194	−15.93	0.058
2.05	0.5	0.25	0.12	0.75	0.194	−15.93	0.058

*a*_*c*_Pe_*c*_	*S*	*a*_*f*_	*a*_*h*_Pe_*h*_	*L*_*o*_/*L*	*ϕ*_*o*_	*B*	*ϕ*_*o*_
0.12	0.25	0.25	0.05	0.4	0.466	−6.865	0.114
0.12	0.5	0.25	0.05	0.4	0.466	−6.865	0.114
2.05	0.25	0.25	0.05	0.75	0.194	−15.93	0.05
2.05	0.5	0.25	0.05	0.75	0.194	−15.93	0.05

(*) The parameters that produce *λ*_*c*,max⁡_ are *a*_*c*_Pe_*c*_ = 0.6 and (*L*_*o*_/*L* = 0.75, *ϕ*_*o*_ = 0.194) for the case given by (19) while (*B* = −10.62, *ϕ*_*o*_ = 0.074) for the case given by (21)

(**) The parameters that produce *λ*_*h*,max⁡_ are *a*_*h*_Pe_*h*_ = 2.14 and (*L*_*o*_/*L* = 0.75, *ϕ*_*o*_ = 0.194) for the case given by (19) while (*B* = −15.93, *ϕ*_*o*_ = 0.05) for case given by (21).

## Nomenclature

(*a*_*h*_, *a*_*c*_, *a*_*f*_): (Hot channel, cold channel, pin) aspect ratio (3); *a*_*f*_ = *d*_*f*_/*L*_*f*_
(Bi_*h*_, Bi_*f*_): (Hot channel, fin) Biot number (Bi_*h*,*f*_ = *h*_*u*,*f*_{*H*_*h*_, *d*_*f*_}/*k*_*h*,*f*_)
(Bi_*h*,*c*_)_eff_: (Hot, cold) channel effective Biot number (39)
(*c*_*ph*_, *c*_*pc*_): (Hot, cold) fluid specific heat [J kg^−1^ K^−1^]
*d*_*f*_: Pin diameter [m]
(*H*_*h*_, *H*_*c*_): (Hot, cold) channel height [m]
(*h*_*h*_, *h*_*c*_): (Hot, cold) flow convection heat transfer coefficient [W m^−2^ K^−2^]
(*h*_*f*_, *h*_*u*_): (Finned, unfinned) convection heat transfer coefficient [W m^−2^ K^−2^]
(*k*_*h*_, *k*_*c*_, *k*_*e*_): (Hot, cold, auxiliary) fluid thermal conductivity [W m^−1^ K^−1^]
(*L*, *L*_*f*_): (Channels, pin) length [m]
*m**: Fin dimensionless thermal length (10c)
*N*_*f*_: Number of pins (8)
(Nu_*h*_, Nu_*c*_): (Hot, cold) flow Nusselt number (Nu_*h*,c_ = *h*_*h*,*c*_ *H*_*h*,*c*_/*k*_*h*,*c*_)
(Nu_*f*_, Nu_*W*_): (Finned, unfinned) auxiliary flow Nusselt number (43)
(Pe_*h*_, Pe_*c*_): (Hot, cold) flow Péclet number (Pe = RePr)
Pr_*h*,*c*,*e*_: (Hot, cold, auxiliary) fluid Prandtl numbers (Pr = *μc*_*p*_/*k*)
(*q*_*h*_′, *q*_*c*_′): Heat transfer rate per unit width in (hot, cold) channel [W m^−1^]
*q*_*o*_′: Heat transfer rate per unit width in conventional exchanger [W m^−1^]
(*q*_*f*_′′)_*h*,*c*_: (Hot, cold) pin base heat flux [W m^−2^]
(*q*_*u*_′′)_*h*,*c*_: (Hot, cold) unfinned surface heat fluxes [W m^−2^]
(Re_*h*_, Re_*c*_): (Hot, cold) flow Reynolds numbers (Re_*h*,c_ = *ρ*_*h*,*c*_ *u*_*mh*,*mc*_ *H*_*h*,*c*_/*μ*_*h*,*c*_)
(Re_*f*_, Re_*W*_): Reynolds number for flow over (unfinned, finned) part (43)
*S*: Dimensionless cold boundary excess temperature (12)
(*T*_*h*_, *T*_*c*_, *T*_*f*_): (Hot fluid, cold fluid, fin) temperature [K]
(*T*_*mh*_, *T*_*mc*_): (Hot, cold) fluid mean bulk temperature [K]
(*T*_*hW*_, *T*_*cW*_): (Hot, cold) boundary temperature [K]
(*U*_*h*_, *U*_*c*_): (Hot, cold) fluid dimensionless axial velocity (*U*_(*h*,*c*)_ = *u*_(*h*,*c*)_/*u*_*m*,(*h*,*c*)_)
(*u*_*h*_, *u*_*c*_, *u*_*∞*_): (Hot fluid, cold fluid, free stream) axial velocity [m s^−1^]
(*u*_*mh*_, *u*_*mc*_): (Hot, cold) fluid mean velocity [m s^−1^]
(*X*_*h*_, *X*_*c*_): (Hot, cold) channel dimensionless axial distance (*X*_*h*,*c*_ = *x*_*h*,*c*_/*L*)
(*x*_*h*_, *x*_*c*_): (Hot, cold) channel dimensionless axial distance [m]
(*Y*_*h*_, *Y*_*c*_): (Hot, cold) channel dimensionless normal distance (*Y*_*h*,*c*_ = *y*_*h*,*c*_/*H*_*h*,*c*_)
(*y*_*h*_, *y*_*c*_): (Hot, cold) channel dimensional normal distance [m]
*W*: Channels width [m].

### Greek Symbols

(*ε*_*h*_, *ε*_*c*_): (Hot, cold) channel effectiveness (26)
(*ϕ*, *ϕ*_*o*_, *ϕ*_*m*_): (Local, average, maximum) pins base area concentration (8), (19)–(21)
(*γ*_*h*_, *γ*_*c*_): (Hot, cold) channel second performance ratio (29)
(*λ*_*h*_, *λ*_*c*_): (Hot, cold) channel third performance ratios (30)
(*μ*_*h*_, *μ*_*c*_, *μ*_*e*_): (Hot, cold, auxiliary) fluid dynamic viscosity [kg m^−1^ s^−1^]
(Θ_*fh*_, Θ_*fc*_): (Hot, cold) dimensionless maximum flux due to conduction (41)
(Θ_*uh*_, Θ_*uc*_): (Hot, cold) dimensionless maximum flux due to convection (42)
(*θ*_*h*_, *θ*_*c*_, *θ*_*f*_): (Hot fluid, cold fluid, pin) dimensionless temperature, (5c), (10b)
(*θ*_*hW*_, *θ*_*cW*_): (Hot, cold) dimensionless boundary temperature (11a), (11b)
(*θ*_*mh*_, *θ*_*mc*_): (Hot, cold) fluid dimensionless mean bulk temperature (17)
(*ρ*_*h*_, *ρ*_*c*_, *ρ*_*e*_): (Hot, cold, auxiliary) fluid densities [kg m^−3^].

### Subscripts

(1, 2): (Inlet, exit)
Max: Maximum value
*o*: Reference case.

## References

[1] Kakac S, Liu H (2002). *Heat Exchangers: Selection, Rating and Thermal Design*.

[2] Hewit GF, Shires CL, Bott TR (1994). *Process Heat Transfer*.

[3] Khaled A-RA, Siddique M, Abdulhafiz NI, Boukhary AY (2010). Recent advances in heat transfer enhancements: a review report. *International Journal of Chemical Engineering*.

[4] Laohalertdecha S, Naphon P, Wongwises S (2007). A review of electrohydrodynamic enhancement of heat transfer. *Renewable and Sustainable Energy Reviews*.

[5] Steinke ME, Kandlikar SG (2004). Review of single-phase heat transfer enhancement techniques for application in microchannels, minichannels and microdevices. *International Journal of Heat and Technology*.

[6] Kay WM, London AL (1984). *Compact Heat Exchangers*.

[7] Saunders EAD (1988). *Heat Exchangers—Selection, Design, and Construction*.

[8] Liu S, Saker M (2013). A comprehensive review on passive heat transfer enhancements in pipe exchangers. *Renewable and Sustainable Energy Reviews*.

[9] Stone KM (1996). A comprehensive review on passive heat transfer enhancements in pipe exchangers.

[10] Bergles AE (1998). *Handbook of Heat Transfer*.

[11] Lee S, Choi SU-S, Li S, Eastman JA (1999). Measuring thermal conductivity of fluids containing oxide nanoparticles. *Journal of Heat Transfer*.

[12] Xuan Y, Li Q (2000). Heat transfer enhancement of nanofluids. *International Journal of Heat and Fluid Flow*.

[13] Hasan MI, Rageb AMA, Yaghoubi M (2012). Investigation of a counter flow microchannel heat exchanger performance with using nanofluid as a coolant. *Journal of Electronics Cooling and Thermal Control*.

[14] Yu W, Xie H (2012). A review on nanofluids: preparation, stability mechanisms, and applications. *Journal of Nanomaterials*.

[15] Kakaç S, Pramuanjaroenkij A (2009). Review of convective heat transfer enhancement with nanofluids. *International Journal of Heat and Mass Transfer*.

[16] Peles Y, Koşar A, Mishra C, Kuo C, Schneider B (2005). Forced convective heat transfer across a pin fin micro heat sink. *International Journal of Heat and Mass Transfer*.

[17] Shafeie H, Abouali O, Jafarpur K, Ahmadi G (2013). Numerical study of heat transfer performance of single-phase heat sinks with micro pin-fin structures. *Applied Thermal Engineering*.

[18] Bejan A (2013). *Convection Heat Transfer*.

[19] Incorpera FP, DeWitt DP, Bergman TL, Lavine AS (2006). *Fundamentals of Heat and Mass Transfer*.

[20] Oosthuizen PH, Naylor D (1999). *Introduction to Convective Heat Transfer Analysis*.

[21] Vera M, Liñán A (2010). Laminar counterflow parallel-plate heat exchangers: exact and approximate solutions. *International Journal of Heat and Mass Transfer*.

[22] Khaled A-RA, Vafai K (2005). Analysis of flexible microchannel heat sink systems. *International Journal of Heat and Mass Transfer*.

[23] Khaled A-RA, Vafai K (2011). Cooling augmentation using microchannels with rotatable separating plates. *International Journal of Heat and Mass Transfer*.

[24] Blottner FG (1977). Finite difference methods of solution of the boundary-layer equations. *The American Institute of Aeronautics and Astronautics Journal*.

